# MALDI-TOF MS Profiling of Annonaceous Acetogenins in *Annona muricata* Products for Human Consumption

**DOI:** 10.3390/molecules14125235

**Published:** 2009-12-15

**Authors:** Pierre Champy, Vincent Guérineau, Olivier Laprévote

**Affiliations:** 1 Laboratoire de Pharmacognosie, CNRS UMR 8076 BioCIS, Faculté de Pharmacie, Université Paris-Sud 11, Rue J. B. Clément, 92296 Châtenay-Malabry, France; 2 Équipe de Spectrométrie de Masse, Institut de Chimie des Substances Naturelles, CNRS UPR2301, 91198 Gif-sur-Yvette, France; E-Mails: vincent.guerineau@icsn.cnrs-gif.fr (V.G.); olivier.laprevote@icsn.cnrs-gif.fr (O.L.)

**Keywords:** *Annona muricata*, annonaceae, annonaceous acetogenin, annonacin, guadeloupean parkinsonism, MALDI-TOF MS

## Abstract

Annonaceous acetogenins are proposed as environmental neurotoxicants consumed through medicinal and alimentary habits and responsible for atypical parkinsonian syndromes observed in tropical areas. Potential sources of exposure still have to be determined, as, to date, only a few batches of products for human consumption were searched for these compounds. To assess the presence of acetogenins, we propose a fast, sensitive and accurate method of screening, using MALDI-TOF MS, with minimal sample preparation. Development of the technique is discussed. Its application to leaves of herbal tea, pulp and bottled nectar of *Annona muricata* is presented.

## Abbreviations

α-CHCA: α-cyano-4-hydroxycinnamate
ACG: Annonaceous acetogenin
amu: atomic mass unit
APCI: atmospheric pressure chemical ionization
DAD: diode array detector
DHB: 2,5-hydroxybenzoyc acid
ESI: electrospray ionization
I.C.: internal calibrant
MALDI-TOF: Matrix-Assisted Laser Desorption/Ionization-Time-of-Flight
MS: mass spectrometry
NMR: nuclear magnetic resonance
RP-HPLC: reversed-phase high performance liquid chromatography
THAP: 2,4,6-trihydroxy-acetophenone
THF: tetrahydrofuran
THP: tetrahydropyran

## Introduction

Ten years ago, *The Lancet* published an article relating the occurrence of a cluster of atypical parkinsonian syndromes in Guadeloupe (French West Indies) [1], where these patients account for two-thirds of all cases of Parkinsonism, compared to approximately 30% in European countries. The disease was thoroughly characterized [2,3,4,5]. Autopsies revealed accumulation of neuronal Tau-fibrils [2,6]. This “Guadeloupean Parkinsonism” was epidemiologically linked to the consumption of plants of the Annonaceae family. Other clusters of atypical Parkinsonism were identified since then, in populations traditionally relying on Annonaceae [7,8,9]. Implication of inhibitors of mitochondrial complex I (NADH-ubiquinone oxydo-reductase) such as 1-methyl-4-phenylpyridinum, paraquat or rotenone in the occurrence of idiopathic Parkinsonism and their use to establish animal models of neurodegeneration have been extensively studied [10]. We showed Annonaceous acetogenins (ACGs) [11,12], such as annonacin (ACG 6, see Figure 3), which are potent inhibitors of the enzyme, to be neurotoxic *in vitro* [13] and *in vivo* [14], in link with Tau [15,16]. Nevertheless, we determined presence of ACGs in leaves tea of *Annona muricata* L., which are of regular medicinal use in the Caribbean. More surprisingly, analysis revealed important concentrations in pulps and processed fruit juices of several edible *Annona* species (*A. muricata*, *A. squamosa* L.) [17,18]. Pomper *et al.* [19] identified three ACGs in the pulp of *Asimina triloba* Dunal., a cultivated Annonaceae of North-America; So did Chen *et al.* [20] and Liaw *et al.* [21] in unripe fruits of *Rollinia mucosa* (Jacq.) Baill. From this co-occurrence of data, the notion that ACGs could be etiological agents for cases of sporadic atypical Parkinsonism and tauopathy worldwide arose. Consequently, to identify sources of exposure (*i.e.*, edible fruits and derived food products, traditional herbal remedies, dietary supplements), sensitive screening methods for unambiguous detection of ACGs are valuable.

ACGs displaying activity towards complex I are preferably extracted with MeOH or CH_2_Cl_2_. For batch to batch comparison in ACGs content, some authors rely on biological testing [2,19,22]. TLC revelation with Kedde reagent indicates presence of most ACGs (sub-type 1, *i.e.*, with an unsaturated γ-methyl-γ-lactone), but not those with saturated lactones. Nevertheless, this method, although practical, lacks specificity and shows poor sensitivity. ^1^H-NMR examination of fractions is commonly used in the course of ACGs purification, based on a search for signals typical of the lactonic ring, but is hardly applicable to crude extracts. HPLC-DAD, because of low specificity at λ = 210 nm, is of limited interest in the absence of standards. RP-HPLC-ESI(+)-MS/MS was successfully used for detection of ACGs in crude samples [23,24,25], but with low precision on mass measurements, and only few exploitable fragments. Dereplication of ACGs containing extracts often proves complex, because of co-elution of these numerous, closely structurally-related, compounds (at least 40-50 ACGs per extract, in our experience).

This prompted us to develop a simple tool for rapid, sensitive and accurate detection of ACGs in low amounts of crude extracts. Matrix-Assisted Laser Desorption/Ionization-Time-of-Flight (MALDI-TOF) MS is applicable to detection of trace compounds in complex mixtures, with low matrixes effects. Sample preparation and acquisition of data require minimal steps. HR measurements are possible [26]. The technique was previously used for quantification of annonacin in samples of *A. muricata*, using an internal standard [17], and for search of this molecule in brain parenchyma of annonacin-treated rats, after extraction and HPLC purification [14]. We here discuss the use of MALDI-TOF for qualitative study of ACGs in crude plant extracts, emphasizing on the choice of working conditions and internal calibrants. Application to several *A. muricata* products is shown. The results have chemotaxonomic and sanitary significance.

## Results and Discussion

### Choice of MALDI-TOF parameters and mass calibration

For ACGs, higher ionic intensities were obtained in the reflectron positive mode, with spectra displaying pairs of [M+Na]^+^ and [M+K]^+^ adducts, in a ratio of approximately 3:2. Adjunction of LiCl or LiI for formation of [M+Li]^+^ adducts, though previously used for structural determination and semi-quantification of ACGs [27,28], did not prove particularly useful in our case. Among the different matrixes tested, THAP (2,4,6-trihydroxy-acetophenone) gave good results, but DHB (2,5-hydroxybenzoyc acid) was preferred despite heterogeneous crystallization using the “dried droplet” method for deposit. Other working conditions and instrumental parameters were also optimized (see Experimental section). Ionic intensities for ACGs of types A (mono-THF) and B (bis-THF) was excellent, with l.o.d. of about 200 fmoles deposited. Peaks resolution was satisfactory enough in these conditions (Rs ~ 6,000-10,000). It is noteworthy that the soft ionization/desorption process in MALDI avoids typical in-source fragmentations observed in ESI or APCI-MS (*i.e.*, losses of H_2_O, CO_2_): Visualized ACGs thus are “native”, and not due to artifactual *m/z* shifts.

**Figure 1:**
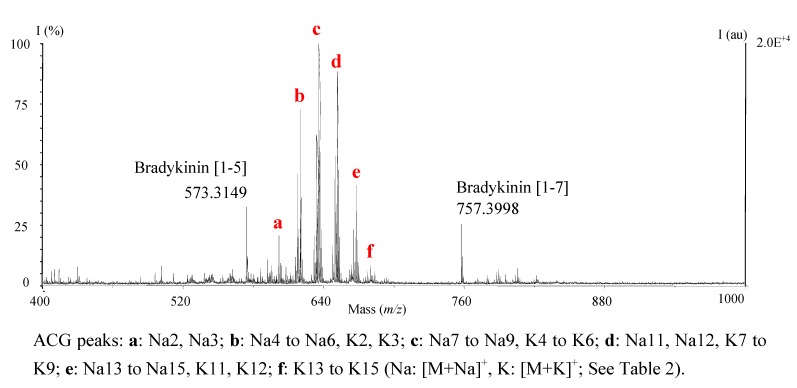
Typical MALDI-TOF spectrum: CH_2_Cl_2_ fraction of aqueous *A. muricata* leaves extract (refluxed H_2_O). *m/z* 400–1,000. I.C.: Pepmix 5.

The following optimization steps were performed with annonacin, a mono-THF tetrahydroxylated ACG of sub-type 1b (C_35_H_64_O_7_), with a methanolic extract of *A. muricata* leaves shown to contain this molecule as major ACG [17], and with combination of both. Internal calibration using matrix peaks (up to *m/z* 275 for DHB) gave unsatisfactory results, with errors on mass measurement of about 40-50 ppm. Internal calibrants (I.C.) of masses closer to that of ACGs (*m/z*: 590-700) were tested: Pepmix 5 (bradykinin [1,2,3,4,5] and [1,2,3,4,5,6,7], *m/z*: 573 and 757), flanks the zone of interest (Figure 1); PEG 400 peaks appear within the mass range of ACGs, with no overlap (Δ_(*m/z* calcd ACG-*m/z* calcd PEG)_ > 0.1 Th). They gave similar results in terms of accuracy (Table 1): For the crude MeOH extract, both modes of internal calibration allowed very satisfactory measurements for [M+Na]^+^ (<5 ppm) and [M+K]^+^ (~15 ppm) adducts of annonacin. However, use of PEG 400 necessitated complicated adaptation of dilutions for adequate intensity in regard to ACGs, peaks of moderate abundance disappearing from spectra when I.C. peaks were too prominent. Nevertheless, calibration step during treatment of data was easier for spectra acquired with Pepmix 5. Using this I.C., deviation on *m/z* values for annonacin peaks, acquired from 15 spectra obtained from three independent experiments, was ± 0.0020 Th (3.2 ppm).

**Table 1:** Comparison of internal calibrants for mass measurement of annonacin (C_35_H_64_O_7_) within a crude MeOH extract of *A. muricata* leaves (**a**) or alone (**b**).

Calibration	[M+Na]^+^			[M+K]^+^		
	*m/z*_calcd_	*m/z*_meas._	error (ppm)	*m/z*_calcd_	*m/z*_meas._	error (ppm)
**a**: Pepmix 5	619.4550	619.4531	3.0	635.4289	635.4382	14.6
**a**: PEG 400		619.4547	0.4		635.4417	20.1
**b**: Pepmix 5		619.4532	3.1		635.4337	7.5

**Table 2:** Acetogenin peaks retrieved in crude extracts of *Annona muricata* derived products.

			**[M+Na]^+^**				**[M+K]^+^**			
Formula	Mass	Source		*m/z*_meas._	*m/z*_calcd_	error		*m/z*_meas._	*m/z*_calcd_	error
C_37_H_66_O_4_	574.4961	p	**Na1 **	597.4801	597.4859	9.6	**K1**	613.4621	613.4598	3.7
		n		597.4792		11.2		613.4510		14.4
C_35_H_62_O_6_	578.4546	*	**Na2**	601.4406	601.4444	6.4	**K2, *Na5***	617.4331	617.4183	23.9
		p		601.4437		1.2		617.4350		27.0
C_35_H_64_O_6_	580.4703	*	**Na3**	603.4512	603.4601	14.6	**K3, *Na6***	619.4530	619.4340	30.6
C_35_H_60_O_7_	592.4340	*	**Na4 **	615.4194	615.4237	6.9	**K4, *Na7***	631.4152	631.3976	27.9
		p		615.4255		3.0		631.4064		13.9
		n		615.4225		1.8		631.4030		8.5
C_35_H_62_O_7_	594.4496	ht	**Na5, *K2***	617.4331	617.4393	10.1	**K5,*Na8***	633.4250	633.4133	18.5
		p		617.4350		6.9		633.4246		17.8
		n		617.4407		2.2		633.4278		22.9
C_35_H_64_O_7_	596.4652	ht	**Na6, *K3***	619.4530	619.4550	3.2	**K6, *Na9***	635.4382	635.4289	14.6
		p		619.4510		6.4		635.4381		14.4
		n		619.4552		0.1		635.4406		18.4
C_35_H_60_O_8_	608.4288	*	**Na7, *K4***	631.4152	631.4186	5.3	**K7**	647.4011	647.3925	13.2
C_35_H_62_O_8_	610.4445	ht	**Na8, *K5***	633.4250	633.4342	14.6	**K8, *Na11***	649.4149	649.4082	10.4
		p		633.4246		15.2		649.4095		2.1
		n		633.4278		10.2		649.4142		9.3
C_35_H_64_O_8_	612.4601	ht	**Na9, *K6***	635.4382	635.4499	18.4	**K9, *Na12***	651.4291	651.4238	8.1
		p		635.4381		18.5		651.4296		9.3
		n		635.4406		14.6		651.4344		16.2
C_37_H_66_O_7_	622.4809	p	**Na10**	645.4689	645.4706	2.6	**K10**	661.4538	661.4446	13.9
C_35_H_62_O_9_	626.4394	ht	**Na11, *K8***	649.4149	649.4292	21.9	**K11 **	665.4069	665.4031	5.8
		n		649.4142		23.0		665.4083		7.8
C_35_H_64_O_9_	628.4550	ht	**Na12, *K9***	651.4291	651.4448	24.1	**K12**	667.4178	667.4187	1.5
		p		651.4296		16.0		667.4142		2.4
C_37_H_64_O_8_	636.4601	*	**Na13**	659.4312	659.4499	28.3	**K13**	675.4328	675.4238	13.2
C_37_H_66_O_8_	638.4758	*	**Na14**	661.4609	661.4655	7.0	**K14**	677.4458	677.4395	9.3
C_37_H_68_O_8_	640.4914	*	**Na15**	663.4749	663.4812	9.5	**K15**	679.4530	679.4551	3.2

Herbal tea (ht), pulp (p) and nectar (n); (*) peaks for refluxed H_2_O leaves extract, in addition to that of (ht); I.C.: Pepmix 5; Error (ppm).

### Treatment of data

Molecular masses of ACGs can be easily predicted: these compounds are constituted of 35 or 37 carbon atoms, apart for few exceptions (short representatives, fatty acid esters). Seven lactonic sub-types are described [11,12] (for convenience, the former classification system for ACG [11] is used in this manuscript. Basically, variations on the alkyl chain reside in number of THF, THP, epoxides or ketones (+13.9792 amu compared to naked carbon backbone), hydroxyl groups (+15.9994 amu), acetyl moieties (+C_2_H_2_O: +42.0106 amu). After calibration, interpretation of data can be achieved using a computer calculation table, and necessitates manual peaks selection. To accelerate this process, we developed a software allowing us to search for pairs of *m/z* values corresponding to [M+Na]^+^ and [M+K]^+^ adducts, with desired maximal error on mass measurement and correct S/N ratio (>10), directly from the peaks list generated by the spectra treatment software. In the following experiments, criteria for precision were that determined for annonacin. It is noteworthy that the presence of isobaric ions leads to isobaric peaks of [C_n_H_m_O_p_+Na]^+^ and [C_n_H_m_O_(p-1)_+K]^+^ species (Δ Na to K: +15.9793 amu). This competition explains the relative differences between experimental and calculated mass values for the most abundant species.

### Study of complex crude extracts

The technique was applied to four CH_2_Cl_2_ extracts, prepared from the following *A. muricata* material:

- Refluxed H_2_O extract of ground leaves (Figure 1);
- Cup of herbal tea prepared from leaves, according to traditional recipe; In this particular case, the amount of CH_2_Cl_2_ extract obtained was low (~1 mg) and ACGs were undetectable with TLC or HPLC-UV, rendering our method particularly useful;
- Lyophilized pulp from Senegal;
- Bottled nectar from Venezuela.

**Figure 2:**
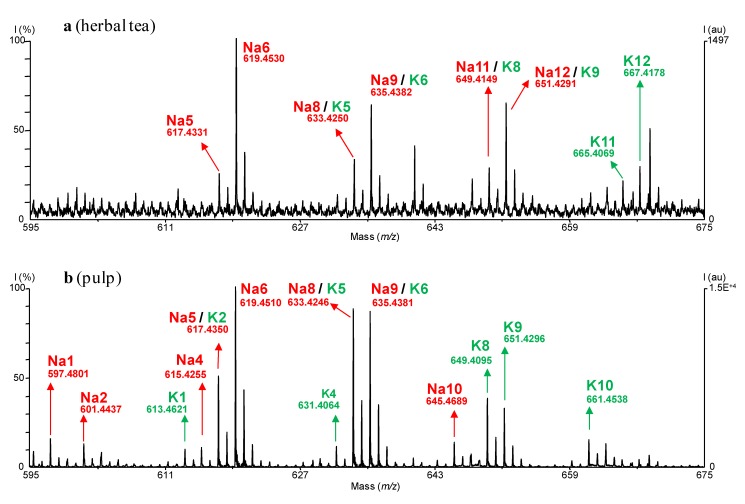
Typical MALDI-TOF spectra: (**a**) CH_2_Cl_2_ extract of herbal tea of *A. muricata*; (**b**) CH_2_Cl_2_ extract of pulp of *A. muricata*. *m/z*: 595–675 (ACG peaks: Na: [M+Na]^+^, K: [M+K]^+^); I.C.: Pepmix 5.

Details of typical spectra are shown in Figure 2a (herbal tea) and 2b (pulp); *m/z* values attributable to ACGs are presented in Table 2.

**Figure 3:**
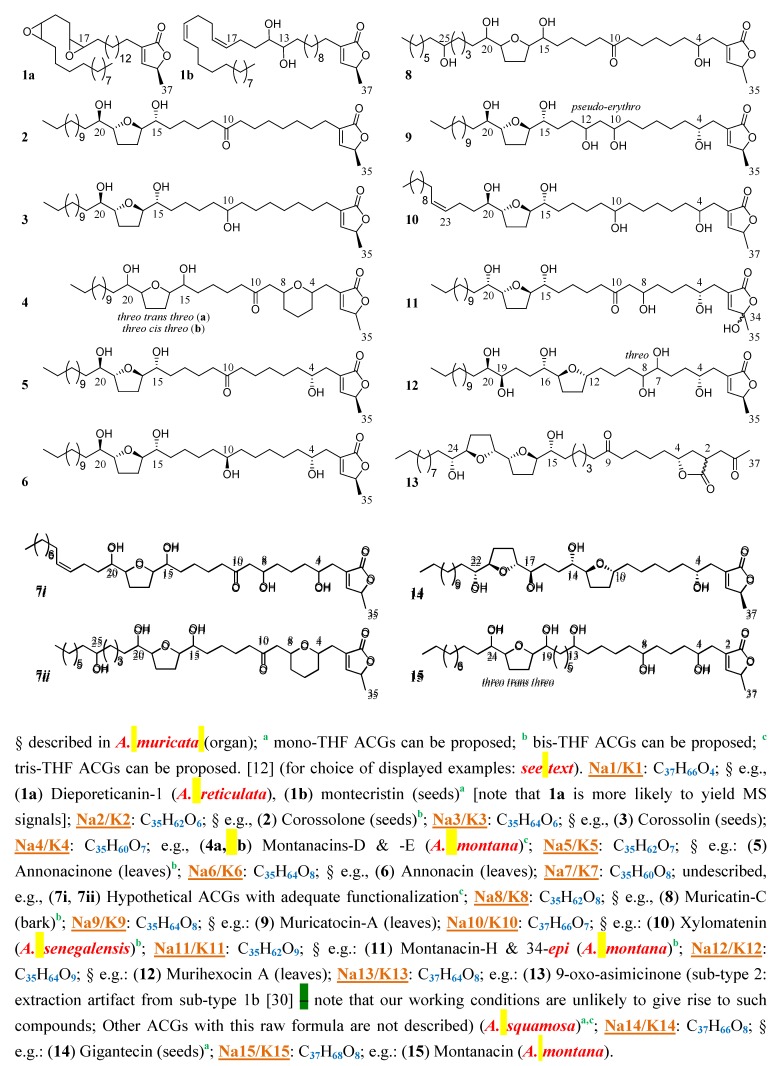
Structures of sub-type 1 ACGs possibly attributable to *m/z* values identified (see Table 2).

For each retrieved mass, numerous described or putative ACGs can be present. Representative structures are given (Figure 3), illustrating structural diversity among the class [12]. These examples were chosen in regard to their relevance, with the following criteria:

- Presence in *A. muricata*; Among ACGs isolated from the species: ~60% bear OH at C-4, ~45% at C-10; ~65% of type A bear an α,α’-diOH-THF system between C-15 and C-20; ~25% of type E (devoid of THF, e.g., **1a**, **1b** in Figure 3) are precursors of the latest [12,29];
- In case of absence in *A. muricata*: Presence in the genus *Annona* [12].

### Interest of the method, chemotaxonomic significance

MALDI-TOF MS evidenced nine, seven and six masses/raw formula corresponding to ACGs in the pulp, nectar and leaves herbal tea, respectively. Seven minor groups of ACGs were observed in refluxed H_2_O leaves extract, in addition to that seen for herbal tea. A leaves crude MeOH extract showed identical qualitative pattern, with similar relative abundances (data not shown). 103 ACGs were isolated from *A. muricata* (bark, fruit, leaf, root and seed) so far [12,29]: ~74% bear 35 carbon atoms and ~65% are of the A1 type (mono-THF, unsaturated lactone). Only one (non-adjacent) bis-THF and no tris-THF ACGs were isolated [11,12]. Most frequently cited raw formulas (C_35_H_64_O_7_, ~20% of ACGs described in the species; C_35_H_64_O_8_, ~20%) correspond to the most abundant groups in our analysis. Even though MALDI lacks quantitation capabilities in the absence of a standard [17], and though isolated compounds do not bear statistical witness of composition, our results are, interestingly, in remarkable agreement with the literature. The major peaks (Na6/K6) thus correspond to those of annonacin, often isolated from the plant, for all three samples [17]. ~25% of ACGs described in the species belong to type E, mostly from seeds, which apolar extracts were extensively studied. Nearly none is visualized (10 un-retrieved peaks, e.g., C_35_H_60_O_5_, [M+Na]^+^: *m/z* = 583.4338, [M+K]^+^: *m/z* = 599.4078, for corepoxylone, a putative precursor of annonacin [12]). Their near absence on spectra might be due to low abundance, low extraction yield and poor desorption rate.

It is also noteworthy that the peak patterns are not identical between the analyzed materials: only five masses/raw formula are shared (peaks Na4/K4 to Na6/K6, Na8/K8, Na9/K9). This is possibly in relation to chemotype and phenotypic discrepancies between organs. Accordingly, four raw formulas for type A1 ACGs found by others are absent, and three were un-described in the species, but identified in other *Annona* spp. (peaks Na4/K4, Na13/K13, Na15/K15). To our knowledge, the raw formula C_35_H_60_O_8_ (peaks Na7/K7) is not described among ACGs [11,12]. Lack of overlap of the [M+K]^+^ adduct (K7) with a [C_35_H_60_O_9_+Na]^+^ adduct rules out any artifactual nature for such compounds.

## Experimental

### General

MALDI-TOF MS was performed with a Perseptive Voyager DE STR MALDI time-of-flight mass spectrometer (Perseptive Biosystems), equipped with a Tektronix TDS 540C digital oscilloscope (500 MHz, digitization rate 2 Gigasamples·s^-1^) and with a N_2_ laser (λ = 337 nm). Extraction solvents were purchased from Carlo-Erba (VWR) and MS solvents from Prolabo. Water was purified by a Millipore water purification system and had a resistivity > 18 MΩ·cm^-1^.

### Plant material and preparation of samples

*A. muricata* fruits were purchased at a market in Dakar (Senegal), seeds and pericarps were removed and pulp was lyophilized, then extracted with CH_2_Cl_2_ (dry mass = 1 g, vol = 50 mL, r.t.; extract: 3.0 mg; plant material was devoid of seed fragments). *A. muricata* bottled nectar from Venezuela (vol: 520 mL; 25% pulp), purchased in a food store in Paris, was diluted to 2 L with H_2_O, then counter-extracted with CH_2_Cl_2_ (1:1 v/v, 2×; extract: 345.4 mg). Aqueous extracts of dried *A. muricata* leaves collected in Martinique (ground leaves 100 g/L, H_2_O 100 °C, 3h, final yield: 0.2%; entire leaves, 2.5 g, infusion in 250 mL H_2_O, 10 min, final extract: 1.3 ± 0.2 mg) were partitioned with CH_2_Cl_2_ (1:1 v/v, 2×). Annonacin was obtained as previously described [14].

### MALDI-TOF experiments and data analysis

Matrixes (α-cyano-4-hydroxycinnamate, α-CHCA; 2,4,6-Trihydroxy-acetophenone, THAP; 2,5-hydroxybenzoyc acid, DHB; Aldrich Chemical Co.) were tested in various solvents and concentrations. Further analyzes were performed with DHB in MeOH/H_2_O (1:1, 20 mg/mL). The samples (10 mg/mL in MeOH or CH_2_Cl_2_) were diluted in matrix solution (1:10, v/v, H_2_O/MeOH 1:1). Deposit on the MALDI plate (1 µL/spot) was done, at least in triplicate, by the “dried droplet method” under atmospheric pressure. MALDI conditions were as follows: mass range: 100-1,000 or 400-1,000; low-mass gate: 80 or 350; laser power: 1,900-1,950 (arbitrary units); accelerating voltage: 20,000 V; grid voltage: 65% of accelerating voltage; delayed extraction time: 100 ns; shots: 200-500/spectrum.

Internal calibration was made by adding Pepmix 5 (1:20, v/v; bradykinin [1,2,3,4,5] and [1,2,3,4,5,6,7], *m/z*_calcd_: 573.3150, 757.3998; LaserBio Labs) or PEG 400 (~1:10,000 v/v, MeOH, *m/z*_calcd_: [M_(n=13)_+H]^+^=591.3592; [M_(n=13)_+K]^+^=613.3411; [M_(n=13)_+K]^+^=629.3150; [M_(n=14)_+H]^+^=635.3854; [M_(n=14)_+Na]^+^=657.3673; [M_(n=14)_+K]^+^=673.3413; [M_(n=15)_+H]^+^=679.4116) to the samples mixed with matrix. Errors on mass measurement [׀(*m/z*_meas._/ *m/z*_calcd_)׀ / *m/z*_calcd_×10^6^] were calculated in parts per million (ppm). For routine analyzes, a typical spectrum is chosen from two to five spectra acquired from each spot. Spectra were analyzed using the Data Explorer software (Perseptive Biosystems) and home-made software created with Windev^®^. For calculations: Na^+^ = 22.9897697 uma, K^+^=38.9637090 uma.

## Conclusions

From these data, only hypothetical structures can be proposed, but MALDI-TOF MS offers valuable advantages: Sample preparation is minimal, desorption conditions are soft, sensitivity and mass accuracy are excellent, acquisition and interpretation of data are rapid (10 min/sample), no internal standard (*i.e.*, purified ACG) is needed, making this technique an interesting screening tool for identification of ACGs in a public health context.
